# A Modified Sequential Deposition Route for High-Performance Carbon-Based Perovskite Solar Cells under Atmosphere Condition

**DOI:** 10.3390/molecules27020481

**Published:** 2022-01-13

**Authors:** Jinyu Wu, Lei Zhang, Qiao Kang, Hongxi Shi, Long Li, Dan Chi, Shihua Huang, Gang He

**Affiliations:** 1 Provincial Key Laboratory of Solid State Optoelectronic Devices, Zhejiang Normal University, Jinhua 321004, China; wjyzjnu@zjnu.cn (J.W.); zhanglei2017@cug.edu.cn (L.Z.); kangqiao@zjnu.edu.cn (Q.K.); SHongxi@zjnu.edu.cn (H.S.); 2 Faculty of Materials Science and Chemistry, China University of Geosciences, Wuhan 430074, China; lilong@cug.edu.cn (L.L.); gh6321@cug.edu.cn (G.H.)

**Keywords:** perovskite solar cell, hole transport material-free, capping layer, crystallization orientation

## Abstract

Carbon-based hole transport material (HTM)-free perovskite solar cells have exhibited a promising commercialization prospect, attributed to their outstanding stability and low manufacturing cost. However, the serious charge recombination at the interface of the carbon counter electrode and titanium dioxide (TiO_2_) suppresses the improvement in the carbon-based perovskite solar cells’ performance. Here, we propose a modified sequential deposition process in air, which introduces a mixed solvent to improve the morphology of lead iodide (PbI_2_) film. Combined with ethanol treatment, the preferred crystallization orientation of the PbI_2_ film is generated. This new deposition strategy can prepare a thick and compact methylammonium lead halide (MAPbI_3_) film under high-humidity conditions, which acts as a natural active layer that separates the carbon counter electrode and TiO_2_. Meanwhile, the modified sequential deposition method provides a simple way to facilitate the conversion of the ultrathick PbI_2_ capping layer to MAPbI_3_, as the light absorption layer. By adjusting the thickness of the MAPbI_3_ capping layer, we achieved a power conversation efficiency (PCE) of 12.5% for the carbon-based perovskite solar cells.

## 1. Introduction

Lead halide perovskites have excellent optoelectronic properties, such as a high absorption coefficient, high carrier mobility, and long carrier recombination life. Due to these excellent properties, the PCE of perovskite solar cells (PSCs) achieved an explosive development from 3.8% to 25.7% in a few years [1,2,3,4,5,6]. Despite the advance in the PCE of PSCs, the moisture instability of the perovskite layer, under ambient conditions, is one of the most urgent problems to be resolved.

As known to us all, the moisture instability of the perovskite layer reduces the lifetime of the solar cells and, thus, limits their application outdoors. Under the combined action of humidity, oxygen, and ultraviolet light, the perovskite active layer will rapidly decompose [7,8,9,10]. Moreover, the majority of PSCs use hole transport material (HTM) to facilitate hole extraction. (*N*,*N*-di-methoxy-phenyl amine)-9,9-spirobifluorene (spiro-OMeTAD) is a common hole transport material in perovskite solar cells, usually doped with bis(trifluoromethane)sulfonimide lithium salt to improve its conductivity. However, lithium salts easily absorb moisture and cause device degradation [11]. The application of HTM also enhances the complexity of device fabrication. Hence, HTM-free PSCs should be investigated. In 2013, Han proposed a full printable HTM-free perovskite solar cell with a carbon electrode [12]. Due to the unique ambipolar property of perovskite, HTM-free perovskite solar cells can obtain a high PCE of 14.3% [13]. Since the hydrophobic carbon electrode can block moisture, the stability of the perovskite solar cell in a humid environment is substantially improved. For the traditional perovskite solar cells, a metal electrode and HTM are needed. However, the metal electrodes are usually deposited by a thermal evaporation process, under high-vacuum conditions, which is a high-cost process. In comparison to metal electrodes, carbon electrodes avoid the phenomenon whereby metal diffuses into the perovskite layer and leads to device degradation. Additionally, carbon electrodes are beneficial to extract holes, eliminating the need to use expensive and unstable HTM, which promotes the commercialized production of low-cost and stable HTM-free perovskite solar cells. As a result, we fabricated the HTM-free perovskite solar cell based on a carbon electrode.

As the hybrid organic–inorganic halide perovskite is sensitive to moisture and oxygen, most of the preparation processes are completed in a glove box [6,14]. Fabricating high-efficiency and stable perovskite solar cells under atmospheric conditions for commercial application is still a huge challenge [15]. It is difficult to prepare a uniform and dense perovskite film under high humidity. Low-quality perovskite films have many holes. Through these holes, the carbon electrode contacts the electron transport layer directly, resulting in carrier recombination and a reduced carrier concentration. Therefore, the gap between quasi-Fermi levels is decreased, which determines the value of the open-circuit voltage (VOC) [16]. In order to reduce carrier recombination, insulating layers were introduced. Cheng used a mesoporous SiO_2_ insulating layer to prepare HTM-free perovskite solar cells in a 50% humidity environment, and achieved an efficiency of 11% [17]. Han et al. proposed a drop-cast method with a mesoporous ZrO_2_ insulating layer to prepare perovskite solar cells with a PCE of 13% under atmospheric conditions [18]. The high-temperature sinter process of the insulating layer increases the cost and energy consumption considerably. The morphology of the perovskite film tremendously depends on the morphology of the PbI_2_ film, so it is key to acquire a uniform and dense PbI_2_ film. Compared to the one-step method, the sequential deposition method has the advantages of controllable perovskite grain size and reproducibility. However, there is the problem that PbI_2_ cannot be completely converted to MAPbI_3_ in a short period of time; for example, converting 200 nm thick PbI_2_ to perovskite completely takes 2 h or longer, while the perovskite will dissolve into the solution in the same amount of time [19]. Hence, exploring new processes for fabricating high-quality and thick perovskite film with no PbI_2_ residue, under atmospheric conditions, is greatly needed.

Here, we propose a modified sequential deposition process to achieve this goal; *N*,*N*-dimethylformamide (DMF) and dimethyl sulfoxide (DMSO) mixture solvent was applied to improve the morphology of the PbI_2_ film. The mixed solvent inhibited the heterogeneous nucleation process of PbI_2_ during the spin-coating process under atmospheric conditions and high humidity, due to the intermediate phase of PbI_2_ (DMSO)_x_, resulting in the formation of uniform and dense PbI_2_ wet films. Subsequently, ethanol solvent treatment was introduced to obtain PbI_2_ films with a unique flaky morphology and a reduced (001) preferred orientation, which could promote the complete conversion of PbI_2_ to perovskite. We obtained perovskite films with different thicknesses by adjusting the thickness of PbI_2_. The experimental results show that the thick active layer can act as a natural insulating layer to block the contact between TiO_2_ and carbon effectively, and decrease the carrier recombination probability. When the thickness of PbI_2_ is 800 nm, the highest open-circuit voltage (968 mV) and highest efficiency (12.5%) were achieved. More importantly, this modified sequential deposition method exhibited good repeatability for the preparation of perovskite film under atmospheric conditions.

## 2. Results and Discussion

**Figure 1:**
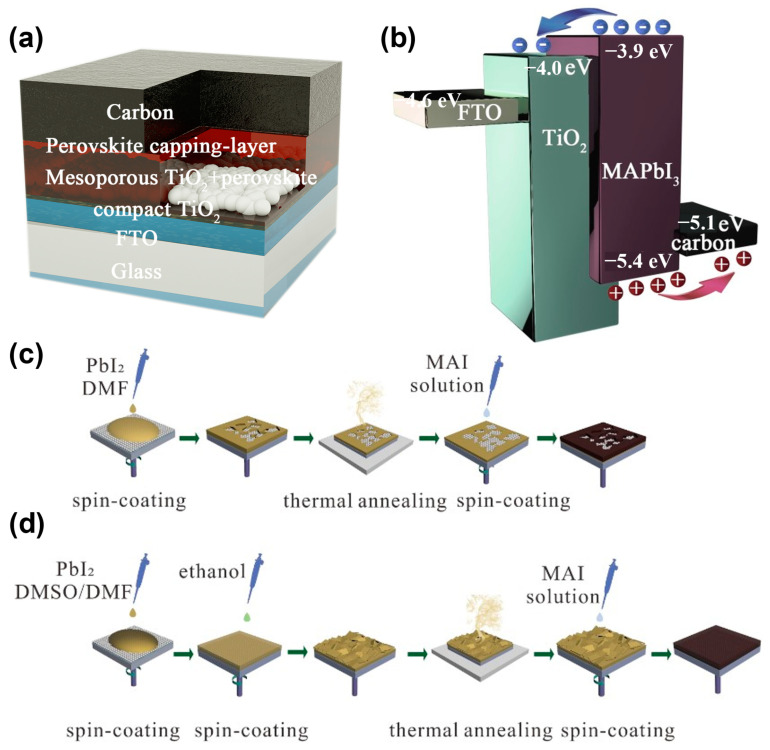
Schematic demonstrating the (**a**) device architecture, (**b**) energy level alignment, (**c**) manufacture process of perovskite thin films by primal and (**d**) modified sequential deposition methods.

The HTM-free perovskite solar cells in this study were structured as fluorine-doped tin oxide (FTO)/compact TiO_2_/mesoporous TiO_2_/MAPbI_3_ capping layer/carbon, as illustrated in Figure 1a. The MAPbI_3_ layers were formed by the sequential deposition method, and the carbon electrodes were prepared using the doctor-blade method with a commercial carbon paste. The band structure alignment of the cell is shown in Figure 1b. We found that the energy levels of these materials match well and the photo-generated carriers can be extracted by the electrodes. Compared with the traditional sequential deposition method, as displayed in Figure 1c, the modified sequential deposition method added DMSO to the PbI_2_ solution, with a 9:1 ratio of DMSO:DMF, and developed the surface engineering by treating the PbI_2_ wet films with ethanol, as illustrated in Figure 1d.

**Figure 2:**
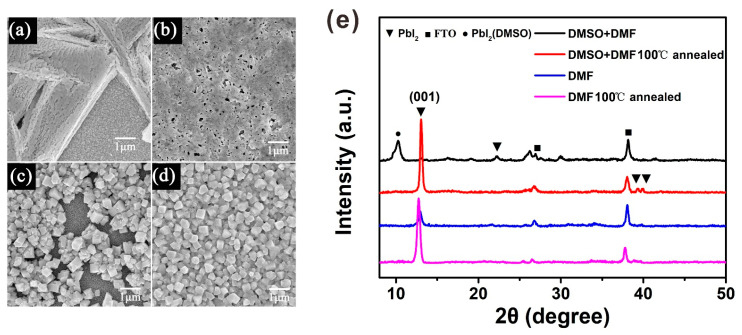
Characterization of PbI_2_ and MAPbI_3_ perovskite films without and with DMSO. (**a**) The top view image of SEM for PbI_2_ film without DMSO. (**b**) The top view image of SEM for PbI_2_ film with DMSO. (**c**) MAPbI_3_ SEM top view image corresponding to PbI_2_ film without DMSO. (**d**) MAPbI_3_ SEM top view image corresponding to PbI_2_ film with DMSO. (**e**) XRD patterns of PbI_2_ films without and with DMSO.

In the sequential deposition process, the quality of the perovskite film was decided by the PbI_2_ film [20]. Cheng et al. found that when the humidity is higher than 20% RH, PbI_2_ tends to exhibit an isolated structure, due to the moisture-induced heterogeneous nucleation and crystallization of PbI_2_ [21]. Our results indicate that the PbI_2_ thin films spin-coated under atmospheric conditions and in high humidity are coarse when using the PbI_2_ solution in DMF, whereas the PbI_2_ thin films are smooth when using the PbI_2_ solution in the DMF and DMSO mixture solvent under the same conditions, as shown in Supplementary Materials Figure S1. In Figure 2a, we can observe that the coarse PbI_2_ thin film exhibits an isolated branching structure, resulting in poor coverage of the MAPbI_3_ thin film. This can be demonstrated by the scanning electron microscopy (SEM) image of MAPbI_3_ in Figure 2c. On the contrary, the smooth PbI_2_ film exhibits the ideal morphology, with a high coverage rate and small pin-holes, as shown in Figure 2b. Therefore, the resulting MAPbI_3_ thin film exhibits a dense-grained uniform morphology, as presented in Figure 2d. Figure 2e shows the XRD patterns of the PbI_2_ films both without and with annealing, which explains the retarded crystallization of PbI_2_ with DMSO during the spin-coating process. Because there are so many diffraction peaks in the XRD figure, and most of them are the same for all the four PbI_2_ films, we use different symbols to distinguish these diffraction peaks from different materials. Without being annealed, the DMF-based PbI_2_ wet film shows an obvious diffraction peak centered at 12.6°, indexing to the (001) crystal plane of PbI_2_. These data imply that parts of PbI_2_ have crystallized in the spin-coating process. However, this diffraction peak was not observed in the PbI_2_ wet film with DMSO, indicating its amorphous feature. After heating the PbI_2_ film at 100 °C for 5 min, it showed a similar XRD pattern to that of the PbI_2_ film with DMF. The DMSO-retarded crystallization mechanism describes how DMSO has stronger coordination ability with PbI_2_ than DMF, and the intermediate state of PbI_2_ (DMSO)_x_ is generated in PbI_2_ solution with mixed solvent. On the other hand, DMSO has a higher boiling point (189 °C) than DMF (152.8 °C), and a lower vapor pressure (0.76 kPa at 60 °C) [19]. Based on these reasons, the spin-coating process of PbI_2_ in mixed solvent describes how the PbI_2_ solution is concentrated with DMF evaporation, and the PbI_2_–DMSO phase is formed simultaneously. DMSO helps to retard the rapid crystallization of PbI_2_ during the spin-coating process, resulting in a uniform PbI_2_ wet film.

**Figure 3:**
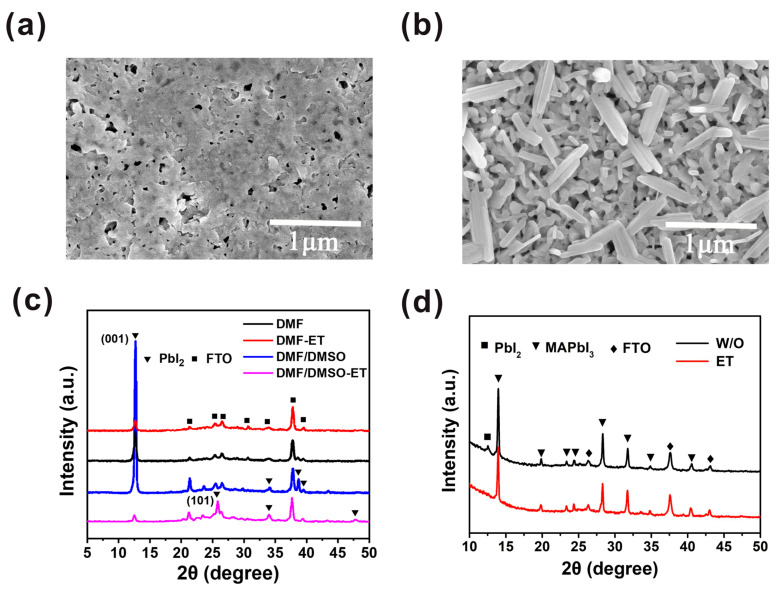
Characterization of PbI_2_ and MAPbI_3_ perovskite films without and with ethanol treatment. (**a**) SEM top view image of PbI_2_ film without ethanol treatment. (**b**) SEM top view image of PbI_2_ film with ethanol treatment. (**c**) XRD patterns of PbI_2_ films without and with ethanol treatment. (**d**) XRD patterns of corresponding MAPbI_3_ films without (black line) and with (red line) ethanol treatment.

In order to improve the morphology of the PbI_2_ film further, the PbI_2_ wet film was exposed to air for tens of seconds, and then the ethanol treatment was introduced. Compared with the PbI_2_ film without ethanol treatment (Figure 3a), we observe that the PbI_2_ film exhibits a porous morphology, with flake-like PbI_2_ crystals, as shown in Figure 3b. The PbI_2_ without ethanol treatment has enough time to nucleate and grow; hence, the PbI_2_ crystal nucleus can grow bigger and hold together. After ethanol treatment, the PbI_2_ film becomes rougher and more porous, thus creating a beneficial condition for PbI_2_ to react with MAI and form MAPbI_3_ film. The morphologies of PbI_2_ and MAPbI_3_ films depend on the exposure time of the PbI_2_ wet film, as displayed in Figure S2. The results confirm that the optimal exposure time is 30 s. After heating the solvent-treated films at 100 °C for 5 min, we note that the diffraction peak intensity at 12.6° of PbI_2_ film based on the mixed solvent and ethanol treatment is much lower than those of the other PbI_2_ films, as presented in Figure 3c. It can be clearly indicated that the crystallization of the PbI_2_ film is lowered by ethanol treatment in combination with mixed solvent. It is known that ethanol can hardly dissolve PbI_2_, but it can separate out the solvent after treating the PbI_2_ film. Therefore, the period for the nucleation and crystal growth of PbI_2_ is restrained, resulting in the decline in PbI_2_ crystallinity. Figure 3c shows that the intensity of the (001) peak of the PbI_2_ film based on the mixed solvent and ethanol treatment is greatly inferior to that of the PbI_2_ film with DMF and ethanol treatment, which results from the higher boiling point of DMSO compared to DMF. Consequently, the nucleation and crystal growth of PbI_2_ in mixed solvent is suppressed. Furthermore, according to the XRD pattern of this reformative PbI_2_ film, the intensity of the (001) peak decreases and the (101) peak appears. This may suggest that the preferred crystallization orientation of PbI_2_ is changed by the ethanol treatment. The XRD spectra (Figure S3) of PbI_2_ with and without annealing have no difference, indicating that PbI_2_ film crystallization was accomplished after ethanol treatment. We attribute the transformation of PbI_2_ crystallization orientation to the changing PbI_2_ growth direction. By annealing the PbI_2_ film, the growth direction of PbI_2_ is altered from a substrate to a PbI_2_ film. In contrast, as we introduce ethanol treatment, the growth direction is altered from a PbI_2_ film to a substrate. Therefore, the preferred orientation of the film is greatly influenced by the substrate composition and roughness [22]. Consequently, the PbI_2_ films generally exhibit (001) lattice plane preferred orientation if traditional annealing is introduced. As we introduce ethanol treatment, the PbI_2_ (001) lattice plane preferred orientation is suppressed, which is attributed to the influence of the released substrate on the PbI_2_ crystallization process.

Previous studies have shown that both reducing the preferred orientation and producing a porous morphology of PbI_2_ are beneficial to realize the full conversion of PbI_2_ to MAPbI_3_ [23]. Under the influence of these two aspects, the solvent-treated film sample (Figure 3d, red line) only showed a series of diffraction peaks of MAPbI_3_. For the sample without the solvent treatment (Figure 3d, black line), the diffraction peak (12.6°) of PbI_2_ still exists, indicative of an incomplete conversion of PbI_2_ to MAPbI_3_. Here, we emphasize that complete conversion is critical for the repeatability of the device. Because the amount of residual PbI_2_ is uncontrollable in an uncompleted conversion perovskite layer, the ratios of MAPbI_3_ to PbI_2_ are different between batches, which decreases the reproducibility of solar cells. Furthermore, photolysis of MAPbI_3_ introduces trap states and reduces the long-term stability of the device [24].

**Figure 4:**
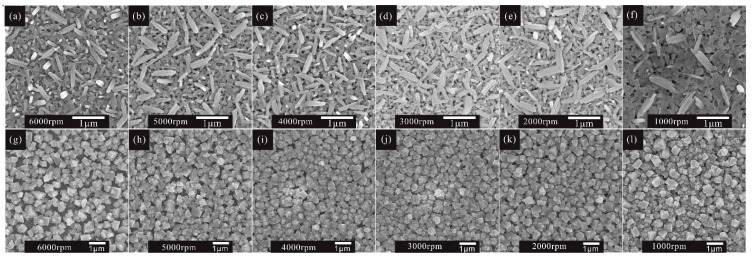
SEM top view images of PbI_2_ films prepared under different spin-coating speeds from (**a**–**f**) 6000 r.p.m. to 1000 r.p.m., and corresponding MAPbI_3_ films (**g**–**l**), respectively.

With the mixed solvent and ethanol treatment strategy, a thick MAPbI_3_ layer without residual PbI_2_ can be obtained. Moreover, the influence of the thickness of the perovskite capping layer on the performance of solar cells was investigated under different spin speeds of PbI_2_, from 6000 r.p.m. to 1000 r.p.m. The perovskite capping layer is the perovskite layer that covers the mesoporous TiO_2_. From the typical cross-sectional figures (Figure S4) of PbI_2_ films, we can observe that the thickness of the PbI_2_ capping layer increases as the rotating speed decreases from 6000 r.p.m. to 1000 r.p.m., and eventually reaches 300 nm. The thickness of the perovskite capping layer follows the same trend as that shown in Figure S5, and can reach 550 nm. It is obvious that the coverage ratio of MAPbI_3_ is also determined by the thickness of PbI_2_ (Figure 4). When the spin speed of PbI_2_ is less than or equal to 3000 r.p.m., a uniform and full coverage MAPbI_3_ on the mesoporous TiO_2_ substrate is obtained, further improving the morphology of the MAPbI_3_ perovskite layer.

**Figure 5:**
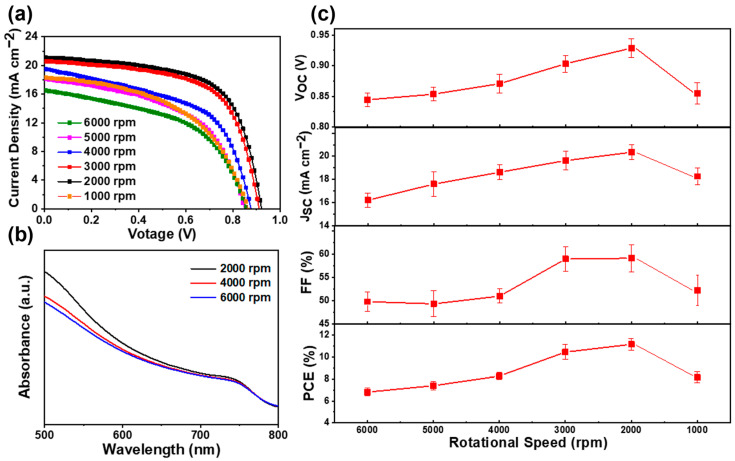
(**a**) J–V curves of perovskite solar cells prepared with PbI_2_ films under different spin-coating speeds from 6000 r.p.m. to 1000 r.p.m. (**b**) UV–vis absorption spectra of MAPbI_3_ films prepared under different spin-coating speeds of 6000, 4000, and 2000 r.p.m. for PbI_2_ films. (**c**) Variations in V_OC_, J_SC_, FF and PCE under different spin-coating speeds from 6000 r.p.m. to 1000 r.p.m.

In order to investigate the influence of perovskite thickness on the performance of solar cells, we fabricated a series of devices with different rotating speeds of PbI_2_. Figure 5a demonstrates the J–V characteristics of the perovskite solar cells under different rotating speeds; their corresponding statistical photovoltaic parameters are shown in Figure 5c and listed in Table 1. The perovskite solar cell fabricated with a 6000 r.p.m. rotating speed of PbI_2_ shows a V_OC_ of 844 mV, J_SC_ of 16.2 mA cm^−2^, FF of 49.8%, and PCE of 6.8%. As the rotating speed of PbI_2_ decreases, all the parameters improve simultaneously. The enhanced PCE is owed to the enlarged J_SC_, V_OC_, and FF, as shown in Figure 5c. As mentioned previously, by using a low rotating speed in the PbI_2_ spin-coating process, the thick MAPbI_3_ capping layers have a better coverage rate than that when using a high rotating speed. It is obvious that the perovskite layer with a better coverage rate can absorb more light and suppress carrier recombination, which is certified by the larger J_SC_ of the device with a lower spin speed of PbI_2_. According to the UV–vis absorption spectra, as shown in Figure 5b, more light can be absorbed by thicker perovskite films, resulting in a larger J_SC_. As we know, the excellent coverage of the perovskite layer is beneficial to separate the carbon electrode from the TiO_2_ layer, thus resulting in a low recombination current and a high V_OC_. Compared to the devices with PbI_2_ spin speeds of 3000 r.p.m. and 2000 r.p.m., we found that the coverage rate of the MAPbI_3_ film was almost similar to that displayed in Figure 4j,k. However, the PSCs with 2000 r.p.m. not only have a larger J_SC_, but also have a higher V_OC_ than those with 3000 r.p.m. The only difference is the thickness of the MAPbI_3_ capping layers. According to these results, we can conclude that increasing the thickness of the capping layer is beneficial to isolate the carbon electrode from the TiO_2_ layer; therefore, the charge recombination between the ETL and the carbon electrode is effectively suppressed. The series resistance (R_S_) and shunt resistance (R_SH_) were calculated using a single-diode model. The attained values of R_S_ and R_SH_ are listed in Table 1. The solar cells fabricated under a low rotating speed of PbI_2_ have larger R_SH_ than those fabricated under a high rotating speed, which was consistent with the improved FF and V_OC_ observed in these solar cells.

The observed decrease in performance for the solar cells fabricated under 1000 r.p.m. is mainly due to the excessive thickness of the MAPbI_3_ layer, which could increase the leakage current of the device, which is further confirmed by the descended J_SC_ and V_OC_ of the devices. When 2000 r.p.m. was applied, a ~500 nm perovskite capping layer was attained. The device fabricated at this spinning speed showed excellent performance. The measured V_OC_, J_SC_, and FF are 929 mV, 20.4 mA cm^−2^, and 59.1%, respectively, which is equivalent to an average PCE of 11.9%.

**Figure 6:**
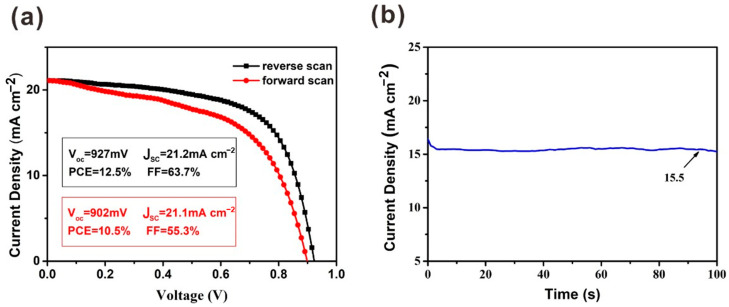
(**a**) J–V curves under reverse scan and forward scan with the preparation of PbI_2_ film under 2000 r.p.m. (**b**) Steady-state output measurements for this device.

Due to the existence of hysteresis, the J–V measurement cannot evaluate the PCE of solar cells exactly. The differences in V_oc_ and J_SC_ are slight with forward scan and reverse scan. However, compared with the parameters under reverse scan, the FF obtained under forward scan is much lower (Figure 6). To precisely evaluate the performance of this device, a steady-state measurement was carried out for the device at the maximum power point (MPP). A J_SC_ of 15.5 mA cm^−2^ was obtained when a bias voltage of 0.75 V was applied. Finally, the calculated PCE from the steady-state output measurement is 11.6%.

## 3. Materials and Methods

### 3.1. Materials

All the materials were purchased from Sigma Aldrich unless otherwise stated. Methylammonium iodide (MAI), commercial carbon paste and FTO-coated glass substrates (Tec15) were purchased from Advanced Election Technology Co., Ltd. (Shenzhen, Guangdong, China)

### 3.2. Preparation of MAPbI_3_ Film

PbI_2_ (556 mg) was dissolved into 1 mL DMF or DMSO and DMF (volume ratio = 1:9) mixing solvent at 70 °C by stirring for 12 h. Figure 1c demonstrates the primal deposition process of the MAPbI_3_ films. We dropped the PbI_2_ solution onto the mesoporous TiO_2_-coated FTO substrate at 6000 r.p.m. for 30 s. The wet PbI_2_ films were annealed at 100 °C for 5 min. Next, 7mg/mL MAI isopropanol solution was loaded onto the substrate of PbI_2_ film for 120 s, and then spun at 2000 r.p.m. for 30 s to remove excess solution. The modified sequential deposition process with mixture solvent and solvent treatment is shown in Figure 1d. Following this, 200 μL ethanol was dripped onto the PbI_2_ wet film and dried using a N_2_ gun after 10 s. The following process is the same as the primal sequential deposition process. The obtained MAPbI_3_ wet films were heated at 100 °C for 30 min.

### 3.3. Device Fabrication

Solar cells were prepared under high-humidity ambient atmosphere. First, FTO-coated glass substrates were cleaned by ultrasonication in detergent, deionized water, acetone and isopropanol for 15 min, respectively. Then, the substrates were dried with a N_2_ gun and subjected to an ultraviolet/ozone cleaning system for 15 min. Subsequently, the titanium diisopropoxide bis(acetylacetonate) solution (75% in 2-propanol, Sigma-Aldrich) diluted in isopropanol (0.15 M) was spin-coated onto the FTO substrates at 4000 r.p.m. for 30 s and annealed at 150 °C for 10 min.

A commercial TiO_2_ paste (Dyesol 18NRT, Greatcell) diluted in ethanol (1:2.5, weight ratio) was dropped onto the compact TiO_2_ and spun at 3000 r.p.m. for 30 s. After drying at 100 °C, the TiO_2_ thin films were annealed at 500 °C for 30 min and then slowly cooled to room temperature. The mesoporous TiO_2_ was treated with a TiCl_4_ aqueous solution (40 mM) at 70 °C for 30 min. After that, the substrate was washed with ethanol and deionized water. The CH_3_NH_3_PbI_3_ film was fabricated using the sequential deposition method. Finally, a counter electrode was prepared by doctor blading the commercial carbon paste onto the perovskite film and annealed at 100 °C for 30 min.

### 3.4. Characterization

The XRD spectra were produced by Bruker D8 Advance X-ray diffractometer (Bruker, Karlsruhe, Germany) with Cu Kα as the radiation source. The top view and cross-sectional SEM images were collected on SU8010 (Hitachi, Tokyo, Japan) and the electron beam was accelerated at 5 kV. Ultraviolet–visible absorption spectra were obtained by PerkinElmer Lambda 35 (PerkinElmer, Waltham, Massachusetts, United States) in the range of 300 to 800 nm at room temperature. Photocurrent−voltage characteristics were obtained on a Keithley 2400 Source Meter under AM 1.5 illumination (Oriel Instruments, Franklin, Massachusetts, United States), which was calibrated by a Si reference cell. The J–V curves were measured by reverse (1.0→−0.1 V) or forward (−0.1→1.0 V) scan, with a scan rate of 50 mV s^−1^. The measured area was confirmed with a metal mask of 0.09 cm^−2^.

## 4. Conclusions

In summary, a simple and effective modified sequential deposition method to fabricate high-quality perovskite film in high humidity was demonstrated. By applying the mixed solvent in combination with ethanol treatment, a thick, uniform and porous PbI_2_ film is formed in the first step. The porous morphology and reduced crystallization orientation of the PbI_2_ film facilitates the diffusion of the MAI solution, and, thus, the PbI_2_ film with a thickness of 800 nm can be completely converted to MAPbI_3_ perovskite in only two minutes. By improving the thickness of the perovskite capping layer, the coverage rate of perovskite is increased further. Obviously, the compact and thick perovskite films strengthen the absorption of incident light, and act as an insulating layer to suppress the carrier recombination between the ETL and carbon counter electrode. Therefore, enhanced photovoltaic parameters are observed. With the optimized thickness of the perovskite layer, the HTM-free carbon-based perovskite solar cell exhibits a superior PCE of 12.5%. In addition, this work provides an excellent approach to fabricating the MAPbI_3_ perovskite layer in air for solar cells and other photoelectric devices.

## Figures and Tables

**Table 1:** Statistics PV parameters of devices processed from PbI_2_ films with different spin-coating speeds.

Spin-Coating Speed (r.p.m.)	V_OC_ (mV)	J_SC_ (mA cm^−2^)	FF (%)	PCE (%)	R_S_ (Ω·cm^2^)	R_SH_ (Ω·cm^2^)
1000	855 ± 17	18.3 ± 0.7	52.2 ± 3.2	8.2 ± 0.5	5.4	249.71
2000	929 ± 15	20.4 ± 0.7	59.1 ± 2.9	11.2 ± 0.5	5.5	357.6
3000	903 ± 14	19.6 ± 0.8	58.9 ± 2.6	10.5 ± 0.7	5.4	330.6
4000	872 ± 15	18.6 ± 0.7	50.0 ± 1.5	8.3 ± 0.3	5.8	186.7
5000	854 ± 11	17.6 ± 1.0	49.3 ± 2.8	7.4 ± 0.4	6.2	166.5
6000	844 ± 11	16.2 ± 0.6	49.8 ± 2.0	6.8 ± 0.4	6.0	165.5

## Data Availability

Data will be available from the corresponding author upon logical request.

## Supplementary Materials

The following are available online, Figure S1: Photograph of PbI2 films with (left) and without DMSO (right). Figure S2: SEM top view images of PbI2 films prepared under different exposure time from (b–f) 10 s to 30 s, and corresponding MAPbI3 films (f–h), respectively. Figure S3: XRD patterns of corresponding MAPbI3 films without (black line) and with annealing (red line). Figure S4: Cross-sectional SEM images of PbI2 films prepared under different spin-coating speeds from (a–f) 6000 r.p.m. to 1000 r.p.m. Figure S5: Cross-sectional SEM images of MAPbI3 films corresponding PbI2 films prepared under different spin-coating speeds from (a–f) 6000 r.p.m. to 1000 r.p.m.

## Sample Availability

Not available.
