# Supplementary material for: A Modified Sequential Deposition Route for High-Performance Carbon-Based Perovskite Solar Cells under Atmosphere Condition
Source: Molecules. 2022 Jan 13;27(2):481. doi: 10.3390/molecules27020481 (PMC8781127; doi:10.3390/molecules27020481)
Supplement: Supplementary file 1 [file molecules-27-00481-s001.zip › molecules-1535664-supplementary.pdf]

A modified sequential deposition route for high-performance carbon-based perovskite solar cells under atmosphere condition

Jinyu Wu[1] , Lei Zhang[1,2], Qiao Kang[1], Hongxi Shi[1], Long Li[1], Dan Chi[1,*], Shihua Huang[1,*], Gang He[2,*]

[1] Provincial Key Laboratory of Solid State Optoelectronic Devices, Zhejiang Normal University, 321004, China;chidan@zjnu.edu.cn, huangshihua@zjnu.cn

[2] Faculty of Materials Science and Chemistry, China University of Geosciences, Wuhan, 430074, China. Email: gh6321@cug.edu.cn

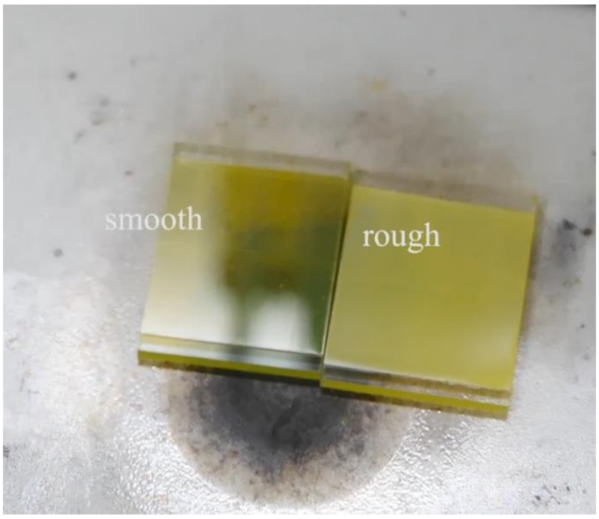

Figure S1. Photograph of PbI_2 films with (left) and without DMSO (right)

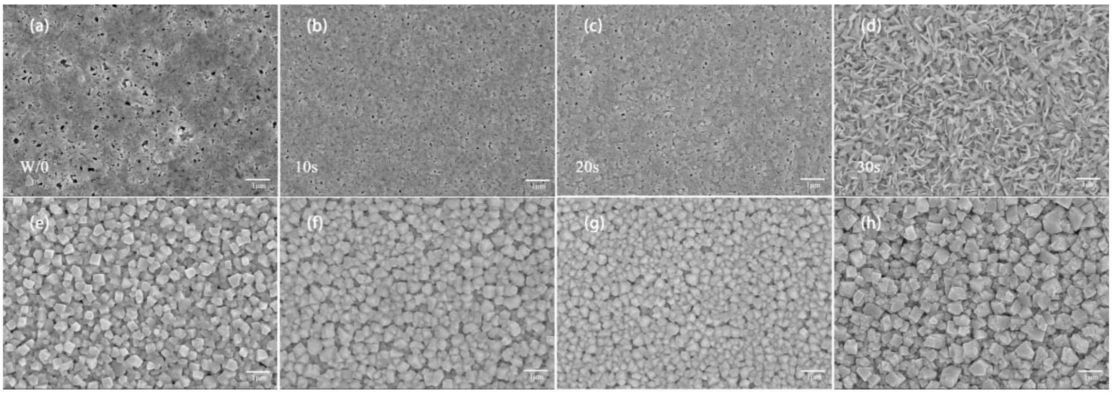

Figure S2. SEM top view images of PbI_2 films prepared under different exposure time from (b)–(f) 10s to 30s, and corresponding $MAPbI_3$ films (f)–(h), respectively.

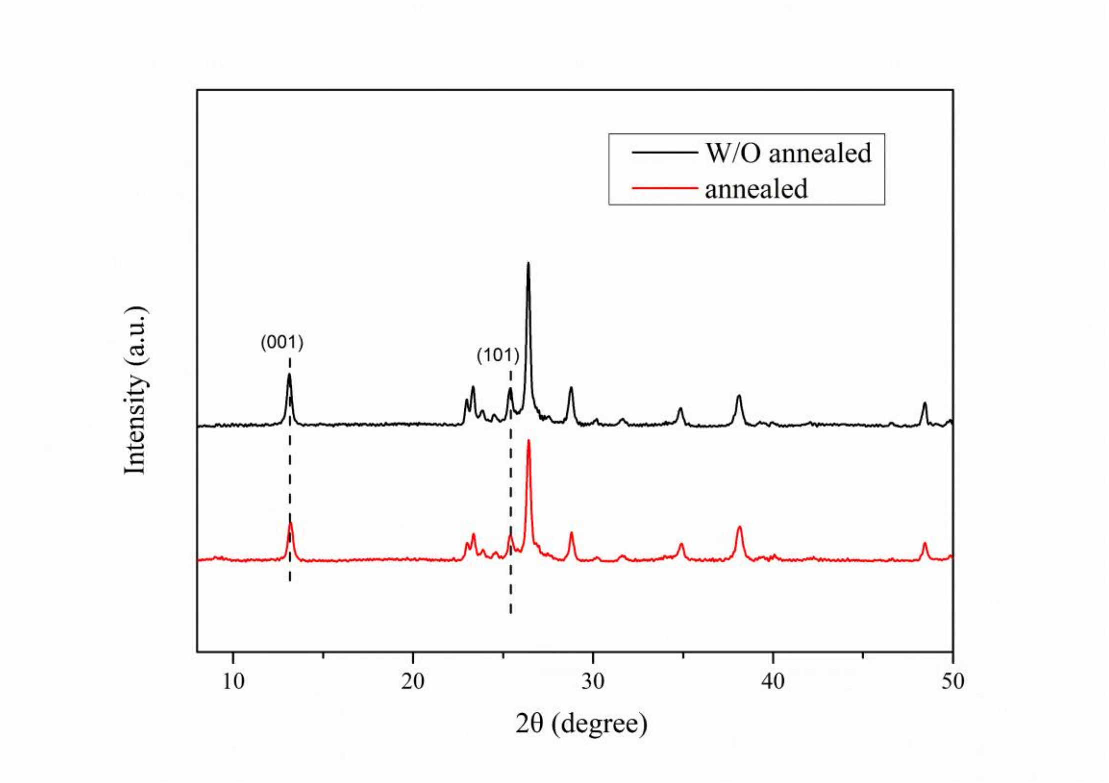

Figure S3. XRD patterns of corresponding $MAPbI_3$ films without (black line) and with annealing (red line).

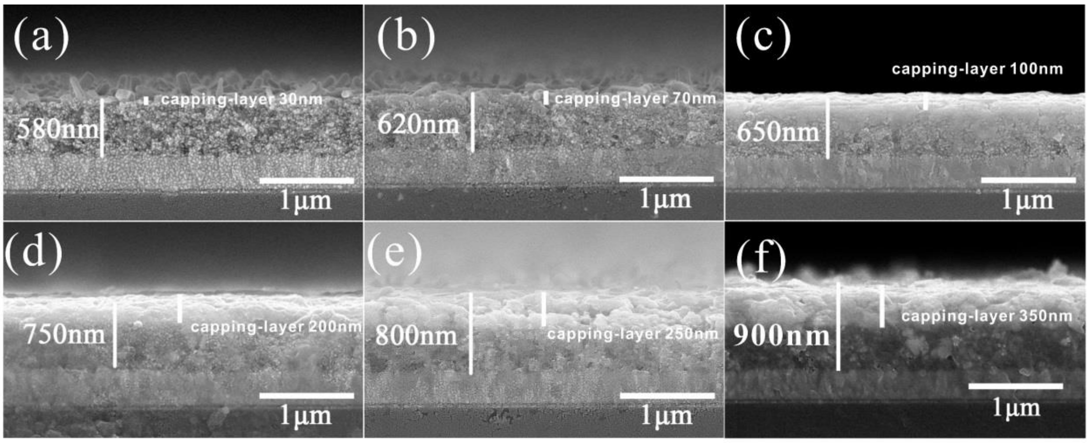

Figure S4. Cross-sectional SEM images of PbI_2 films prepared under different spin-coating speeds from (a)–(f) 6000 r.p.m. to 1000 r.p.m..

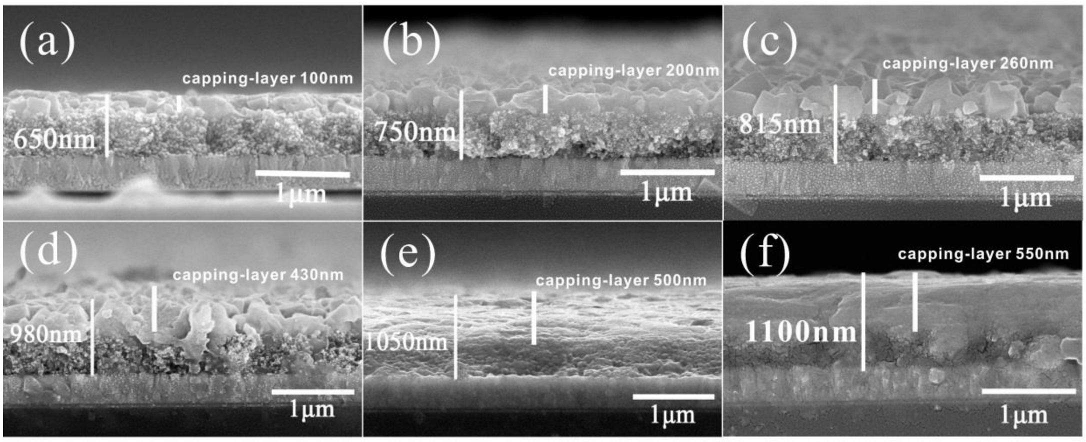

Figure S5. Cross-sectional SEM images of $MAPbI_3$ films corresponding PbI_2 films prepared under different spin-coating speeds from (a)–(f) 6000 r.p.m. to 1000 r.p.m..
